# Thermal traits govern the response of microbial community dynamics and ecosystem functioning to warming

**DOI:** 10.3389/fmicb.2022.906252

**Published:** 2022-08-17

**Authors:** Francisca C. Garcia, Ruth Warfield, Gabriel Yvon-Durocher

**Affiliations:** ^1^Environment and Sustainability Institute, University of Exeter, Penryn, Cornwall, United Kingdom; ^2^Red Sea Research Center, King Abdullah University of Science and Technology, Thuwal, Saudi Arabia

**Keywords:** microbes, temperature, traits, diversity, community structure, ecosystem functioning

## Abstract

Understanding the ecological processes that underpin the dynamics of community turnover in response to environmental change is critical to predicting how warming will influence ecosystem functioning. Here, we quantify the effect of changing temperature on community composition and ecosystem functioning *via* the action of ecological selection on population-level thermal traits. To achieve this, we use microbes isolated from a network of geothermal streams in Iceland where *in situ* temperatures span 8–38°C within a single catchment. We first quantified variability in thermal tolerance between taxa, and then assembled synthetic communities along a broad thermal gradient to explore how temperature-driven selection on thermal tolerance traits shaped the emergent community structures and functions. We found marked changes in community structure and composition with temperature, such that communities exposed to extreme temperatures (10, 35°C) had highly asymmetric biomass distributions and low taxonomic richness. Thermal optima were a good predictor of the presence and relative abundance of taxa in the high-temperature treatments. We also found that the evenness of the abundance distribution was related to ecosystem production, such that communities with more equitable abundance distribution were also the most productive. Our results highlight the utility of using a multi-level approach that links population-level traits with community structure and ecosystem functioning to better understand how ecological communities will respond to global warming.

## Introduction

Habitat destruction, over-exploitation, and non-native species invasions have been the prominent focus of research into biodiversity loss, but climate change is predicted to emerge as the main threat to global biodiversity over the coming decades (Ceballos et al., 2015; Cowles et al., 2016; Craven et al., 2016; Newbold, 2018). Currently, an estimated 25% of all species are at threat from extinction and that number is projected to rise further in the future (Ceballos et al., 2015, 2017). Substantial evidence suggests that the structure of ecological communities (e.g., taxonomic composition, species richness, and biomass distribution) is critical to maintaining the stability and productivity of ecosystems and that the reorganization of community structure due to environmental change will compromise the capacity of ecosystems to provide vital services (e.g., carbon storage and maintenance of soil fertility; Loreau et al., 2003; Elmqvist and Fragkias, 2013; Awasthi et al., 2014; Garcia et al., 2018; Garcia-Palacios et al., 2018). Understanding how novel communities emerge under global warming is therefore crucial to predict the impact on ecosystem functioning (Angilletta, 2009; Isbell et al., 2015; Garcia et al., 2018; Bestion et al., 2020).

Traits are measurable physiological or morphological properties of individuals that strongly influence organism performance and ultimately fitness (Lavorel and Garnier, 2002; Violle et al., 2007). Different traits (or combinations of traits) affect fitness under particular environmental regimes, and therefore, phenotypic trait variance is thought to be a key feature that determines how ecosystem structure and function respond to environmental change (Norberg et al., 2001; Cunningham and Read, 2003; Enquist et al., 2015). Trait-based approaches have received growing interest because they offer the potential of developing a predictive framework for understanding of how species organize within communities and indeed how they may reorganize under environmental change (Lavorel and Garnier, 2002; McGill et al., 2006; Litchman and Klausmeier, 2008).

**Figure 1:**
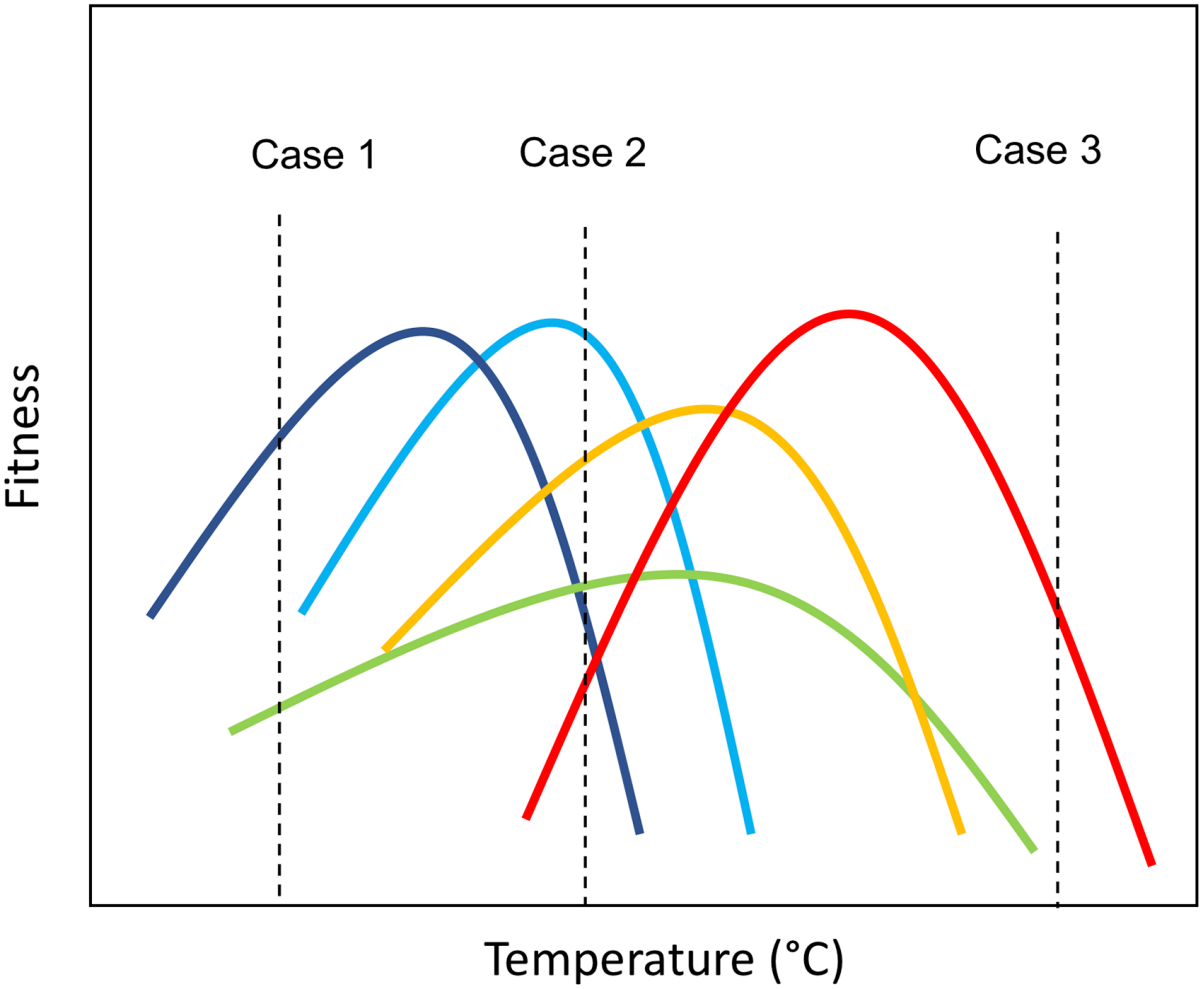
Graphical representation of community assembly based on thermal traits under different temperature conditions. Case 1: Community exposed to cold temperature (~10°C); Case 2: Community exposed to ambient temperature (~20°C); and Case 3: Community exposed to warm temperature (~40°C). The vertical axis represents the fitness of the species (e.g., growth rate). Consider, for example, a pool of species with diversity in their thermal tolerance traits, if the species pool is exposed to different thermal scenarios, the taxonomic structure of the assemblage may differ based on the relative fitness of the species’ embodied in their tolerance curves. At benign temperatures where few species experience thermal stress, communities are likely to have high levels of species richness (Case 2). However, under thermal stress, due to either low (case 1) or high temperatures (case 3), communities are likely to move toward being dominated by a few taxa that are able to cope with the (relatively) extreme environmental conditions. These communities are characterized by low species richness and low evenness in the distribution of biomass among species, i.e., the few species that can survive the extreme conditions dominate the community.

Thermal performance curves (TPC) characterize how components of fitness (e.g., individual growth rate) change with temperature and have a unimodal shape, increasing up to a maximum (optimal temperature, *T*_opt_) and then rapidly declining (Angilletta, 2009; Padfield et al., 2016). The parameters describing the shape of the curve (e.g., *T*_opt_) can be considered traits—i.e. characteristics that influence fitness under environmental variation (Schaum et al., 2017; Bestion et al., 2018; Kontopoulos et al., 2020). These traits are likely to influence both species’ persistence in the environment as temperature regimes change and their contribution to ecosystem functioning by shaping performance relative to competitors (e.g., in terms of growth rate and biomass production; Norberg et al., 2001; Cunningham and Read, 2003; Garcia et al., 2018). Thus, species-specific thermal tolerance traits offer a tool to understand (and even predict) how novel communities may emerge as ecosystems warm in the coming decades. Consider, for example, a pool of species with diversity in thermal tolerance traits, if the species pool is exposed to different thermal scenarios, the taxonomic structure of the assemblage will be influenced by the differential performance of the species embodied in their tolerance curves (Figure 1). At benign temperatures where few species experience thermal stress, temperature will have little impact on community structure, which will be determined by other limiting factors (resource competition, predation, and parasitism; Figure 1; Case 2). However, under thermal stress, due to either low (case 1) or high temperatures (case 3), communities are likely to move toward being dominated by a few taxa that can cope with the (relatively) extreme environmental conditions. These communities are characterized by low species richness (Figure 1) and low evenness in the distribution of biomass among species, i.e., the few species that can survive the extreme conditions dominates the community. Consequently, declines in species richness and shifts toward more asymmetric biomass distributions are anticipated to significantly impair ecosystem function (Norberg et al., 2001; Litchman et al., 2007; Enquist et al., 2015).

Trait-based approaches have been used to understand a wide variety of taxa (plants, phytoplankton, zooplankton, and microbial communities). These studies are typically conducted at broad scales using observational data and often within a single trophic level (Westoby and Wright, 2006; Follows et al., 2007; Follows and Dutkiewicz, 2011; Edwards et al., 2013; Record et al., 2013; Coles et al., 2017). While observational studies have led to major advances in the development of trait-based approaches, they also suffer from limitations because environmental variables that are hypothesized as being key drivers of variation in traits and fitness are often confounded, making it challenging to causally link traits with the environment. By contrast, experimental approaches offer the opportunity to test causal relationships between environmental factors and the distribution of traits in communities, and their effect on ecosystem functioning. Of course, the drawback of an experimental approach is that experiments are often conducted in highly simplified environments that can be limited in terms of their ecological realism. While observational studies in traits-based ecology have been plentiful, experimental approaches have received far less attention as a tool for developing and testing trait-based theory (Elmendorf et al., 2012, 2015; Khaliq et al., 2014; Pinsky et al., 2019).

In this regard, microbial communities are an excellent model system for investigating the link between species traits and community structure because some traits can be easily quantified, ecological dynamics play out rapidly and experiments can be carried out in high throughput with massive replication (Bell et al., 2005; Fiegna et al., 2015; Goldford et al., 2018). Microbes also play a key role in ecosystem processes such as the decomposition of organic matter, the recycling of nutrients as well as forming the base of food webs (Woodwell et al., 1998; Woodward, 2007). Disruption to microbial community structures could therefore affect the structure and functioning of entire ecosystems (Yvon-Durocher et al., 2010; Wang et al., 2014). Here, we use bacterial isolates from freshwater Icelandic geothermal streams to investigate how species richness (the number of species) and evenness (the relative abundance of species) respond to changing thermal regimes and how these changes may affect ecosystem functioning. We also investigate how variance in thermal tolerance traits between taxa shape compositional changes in the communities under different thermal regimes. These experiments aim to further improve our understanding of the mechanisms that drive community responses to environmental warming to ultimately help build a body of knowledge that can be used to develop models that can predict the impacts of environmental change on ecosystem services.

## Materials and methods

Biofilm samples were collected in May 2016 from the surface of rocks from 11 natural freshwater streams in Hveragerdi Valley, Iceland (latitude = 64.02, longitude = −21.18). These groundwater-fed streams ranged in temperature from 7 to 38°C due to varying geothermal warming of the bedrock (O’Gorman et al., 2014). Samples were frozen after collection with 17% glycerol and stored at −20°C until they were processed in the laboratory.

Once in the laboratory, samples were thawed at 20°C and were prepared by spreading 10 μl consecutive dilutions onto agar plates with sterile glass beads. The plates were then incubated at 20°C for 10 days to allow the bacterial populations to grow. The resulting colonies were selected at random, placed into 200 μl LB broth, and incubated for 48 h. Samples were subsequently centrifuged, the supernatant removed, and the pellet re-suspended in a mix of LB broth and 17% glycerol before being frozen at −80°C. Isolates were assigned taxonomic identification using 16S PCR followed by Sanger sequencing within the 16S rRNA gene. Taxonomy was classified by using the Silva database. A total of eight different taxa were selected for the experiments based on the ability for visual discrimination of taxa on agar plates. The taxa isolated represented eight different genera and were *Pseudomonas* sp., *Chromobacterium* sp., *Iodobacter* sp., *Serratia* sp., *Aeromonas* sp., *Mucilaginibacter* sp., *Herbaspirillum* sp., and *Janthinobacterium* sp. (details and accession numbers are given in Supplementary Table S1).

### Taxon-level thermal tolerance

The eight taxa were defrosted in LB and acclimated for 24 h. Samples were then transferred into a protozoan media prepared by adding protozoan pellets to autoclaved Volvic® mineral water at a ratio of 0.76 g of pellets to 100 ml of water. The Volvic water is characterized by its low mineralization content and stable pH. For this reason, it is media widely used in microbial experiments. Protozoan pellets were used as they are created from plant material that encompasses a large diversity of carbon sources that facilitate bacterial growth (Tan et al., 2012). Samples were then diluted to a common density and pipetted into a 96-well plate with 200 μl of filtered and autoclaved protozoan medium. Six replicates of each taxon were exposed to nine temperature treatments (0, 15, 20, 25, 30, 35, 40, 45, and 50°C). Furthermore, four “blanks” were included in the well-plate, which solely comprised the protozoan medium. Each well-plate was placed into a Percival incubator at the nine assay temperatures. The optical density (OD_600_) of each replicate was measured every 2 h using a BioTek reader synergy 2 at 600 nm wavelength. The optical density of each replicate was compared to blank samples to account for any organic matter present in the protozoan medium. This process was repeated until each of the taxa reached carrying capacity. Growth rates [*r* (h^−1^)] and carrying capacities were calculated by fitting the logistic growth equation to the biomass measurements using non-linear least squares regression:


(1) $$N_{t}=\frac{K}{1+Ae^{-rt}};A=\frac{K-N_{0}}{N_{0}}\;$$

where *N*_t_ is the biomass at time, *t*, *K* is the carrying capacity, *N*_0_ is the biomass at the start of the experiment, and *r* is the exponential population growth rate (h^−1^). The thermal tolerance curve was calculated by fitting the Sharpe-Schoolfield equation to the average population growth rate (the mean of six technical replicates was calculated at each temperature along the thermal gradient) for each taxon:


(2) $$\begin{array}{c} \ln\left(r\left(T\right)\right)=E_{a}\left(\frac{1}{kT_{C}}-\frac{1}{kT}\right)+\ln\left(r\left(T_{c}\right)\right)\\ \;-\ln\left(1+e^{E_{h}\left(\frac{1}{kT_{h}}-\frac{1}{kT}\right)}\right) \end{array}$$

where *r*(*T*) is population growth rate (h^−1^), *k* is Boltzmann’s constant (8.62*10^−5^ eV K^−1^), *E*_a_ is the activation energy and indicates the steepness of the slope of the rising part of the TPC, *T* is the temperature in Kelvin (K), *E*_h_ is the temperature-induced inactivation of growth above *T*_h_, which is the temperature where half the enzymes are non-functional, and *r*(*T_c_*) is the rate of growth at a reference temperature, in this case, 18°C. Equation 2 was fitted to the growth rate data. Non-linear curve fitting of growth rate data was achieved by modeling 1,000 random sets of initial parameters extracted from a uniform distribution and retaining the combination that returned the lowest Akaike information criterion (AIC score) using the “nlsLoop” package (Padfield et al., 2016) in R program (R Core Team, 2019). The optimum temperature of growth (*T*_opt_), the temperature at which the species shows their maximum growth rate, was calculated by differentiating Equation and solving for the maxima yielding the following equation:


(3) $$T_{\mathrm{opt}}=\frac{E_{h}T_{h}}{E_{h}+kT_{h}\ln\left(\frac{E_{h}}{E_{a}}-1\right)}$$

The different parameters, *T*_opt_, *E*_a_*, r*(*T*_c_), and *T*_h_, that characterize the shape of the TPC are considered hereafter as thermal traits (see estimated parameters, CI, and quasi r^2^ estimates in Supplementary Table S2).

### Quantifying temperature-driven changes in community structure

The eight taxa were defrosted in LB for 24 h at 20°C and then assembled into communities at equal densities. To build the communities, 500 μl of each of the eight taxa was added to 96 ml of 0.2 μm filtered and autoclaved protozoan medium to yield 100 ml of the mixed community sample at each temperature. Four milliliter of each community sample was then placed into 5 ml vials with a 1 ml headspace. This process was repeated 20 times and included four blanks that solely comprised the protozoan medium. The samples were then placed into Percival incubators for 7 days at six temperatures (10, 15, 20, 25, 30, and 35°C). 200 ul of each sample was collected every day so the optical density of each replicate could be quantified using the BioTek reader synergy 2 at 600 nm wavelength. The 200 μl of the sample taken was immediately replaced with 200 μl of Milli-Q water acclimated at the relevant treatment temperature.

Biomass (OD_600_) was monitored every 2 days at each assay temperature during the experimental incubation to assess the changes in biomass over time at each temperature treatment (10, 15, 20, 25, 30, and 35°C). Communities were initially inoculated at low density and reached carrying capacity within 48 h. Biomass then declined logarithmically over the course of the experiment, meaning that the relative change in biomass decelerated over time (Supplementary Figure S3). After 7 days, the change in biomass over time was small for most experimental units, indicating that the community biomass had stabilized.

The changes in biomass over time at different temperature treatments were analyzed using a linear mixed-effect model using R function “lmer” from R package “lme4,” (Bates et al., 2014), treating “replicate” (20 community replicates per temperature) as a random effect on the intercept to account for the non-independence of community replicates with time. Biomass was included in the model as the response variable, time as a predictor variable, and temperature treatment as a factor with six levels. We performed model selection using likelihood-ratio tests starting with the most complex model and sequentially removing terms until all parameters were significant at *p* < 0.05 (see model selection in Supplementary Table S10).

At the end of the incubation experiment, community samples were then diluted depending on the optical density measurements and 10 μl of each sample was plated on agar, (including the blank samples) to a dilution of 10^−5^. These plates were incubated at the relevant temperature treatments until individual colony morphology could be distinguished. Each plate was subsequently photographed, and the microbial colonies were identified using observations of colony appearance.

The colony counts for each replicate at the end of the experiment were used to calculate the relative abundance of each species within their communities and the species diversity, which was quantified using Shannon’s diversity index (*H′*):


(4) $$H^{\prime }=-\overset{R}{\underset{i=1}{\sum }}p_{i}\ln\left(p_{i}\right)$$

where *R* is species richness and *p_i_* is the species richness divided by the total number of individuals found within the community. We also estimated species evenness using Pielou’s evenness (J):


(5) $$J=H^{\prime }/\log\left(R\right)$$

To quantify the effect of temperature on community diversity (richness and evenness), we fitted the metrics of richness and evenness to linear and quadratic models to assess the form of the relationship between diversity and temperature change. Model comparison was conducted *via* sequential likelihood-ratio tests (see model selection in Supplementary Table S3).

To determine whether community composition differed among temperatures, we performed a principal components analysis (PCA) using the taxon-level relative abundance data for each community. Analyses were performed using the R package “vegan” (Oksanen et al., 2012). A permutational multivariate ANOVA (PERMANOVA) was then conducted to test whether the composition of communities differed among temperature treatments using the “adonis” function. A pairwise PERMANOVA was used to assess pairwise comparisons of composition between temperature treatments. This statistical test provides an adjusted value of *p*, which uses the Bonferroni correction to reduce Type 1 error from multiple comparisons. All the analyses were conducted in R program (R Core Team, 2019).

### Linking thermal traits to relative abundance

We explored the coupling between the abundance of a taxon at each temperature and its thermal tolerance traits. We quantified two different traits: species-specific thermal optima (*T*_opt_,°C) and growth rate *r*(T) (h^−1^) at each assay temperature. We used two separate analyses of covariance (ANCOVA) models to test whether relative abundance was influenced by taxon-specific thermal traits [*T*_opt_ or *r*(T)]. For analysis one, we used relative abundance as the response variable, with taxon-specific thermal optima (*T*_opt_) as a continuous covariate and assay temperature as a categorical factor with six levels. For analysis two, we used relative abundance as the response variable, with taxon-specific growth rate at the assay temperature [*r*(T)] as a continuous covariate and assay temperature as a categorical factor with six levels. To quantify the significance of differences in the parameters among all pairwise combinations of the six temperature levels, we used *post-hoc* Tukey’s tests *via* the “glht” function in the “multcomp” package for R statistical software (see Supplementary Tables S5, S6). We only included data for taxa that were present in more than 25% of the replicate communities at each temperature to avoid rare taxa driving the outcomes of the statistical analyses. In this case, only taxa that were frequently observed at each assay temperature contribute to the observed outcomes.

### Impact temperature and community structure on ecosystem function

We quantified whether the ecosystem functioning community was associated with changes in temperature and community structure along the thermal gradient. We considered biomass production (measured as OD_600_) by day 7 of the experiment, as an estimate of ecosystem function (Cardinale et al., 2007; Garcia et al., 2018). We conducted two analyses. The first, assessed relationship between ecosystem function and temperature. The second, assessed relationship between ecosystem function and community evenness. For both analyses, we fitted a polynomial model where ecosystem function was the response variable and either temperature or evenness was continuous predictor variables which included both linear and quadratic coefficients. We then sequentially removed the quadratic and linear coefficients to assess whether a quadratic, linear, or no relationship best characterized the data. Model comparison was conducted *via* sequential likelihood-ratio tests (see model selection in Supplementary Table S7).

## Results

**Figure 2:**
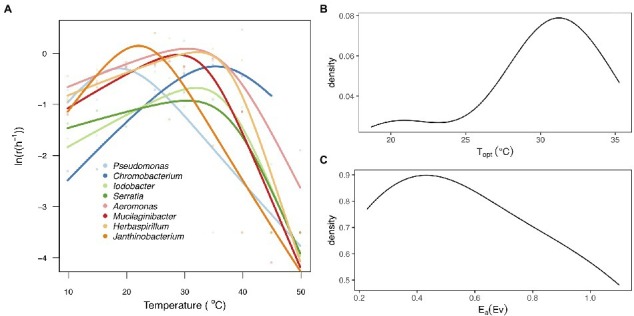
Thermal tolerance curves for the eight bacteria taxa. **(A)** Fitted thermal tolerance curves of population growth rate [ln(*r*(T))] for each of the eight taxa analyzed using the Sharpe-Schoolfield equation (Materials and methods). Different colored lines represent the taxa and different colored points represent the mean values of the technical replicates per taxa and temperature. **(B)** Probability density plot representing the distribution of thermal optimum temperature (*T*_opt_). **(C)** Probability density plot showing variation in the activation energy (*E*_a_) between the eight taxa.

The thermal tolerance curves for each taxon exhibited a characteristic unimodal curve as growth increased exponentially to an optimum and then declined rapidly as temperature increased beyond the optimum (Figure 2A). There was substantial variation among taxa in all the parameters of the thermal tolerance curves (Figure 2; Supplementary Table S2). We found that the *T*_opt_ for growth rate ranged from 18.7°C (*Pseudomonas* sp) to 35.3°C (*Chromobacterium* sp), with a mean of 28.7 ± 5.21°C (Figure 2B), while the *E*_a_ ranged between 0.23 and 1.06 with a mean of 0.58 ± 0.31 (Figure 2C).

**Figure 3:**
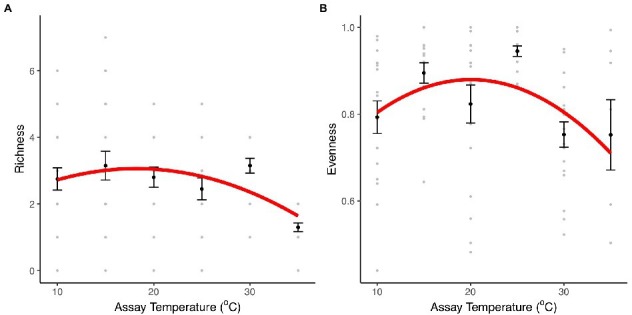
Diversity and richness indices. Species evenness and richness indices for communities comprising eight bacterial taxa incubated at 10, 15, 20, 25, 30, and 35°C. **(A)** Taxon richness for community cultures at each temperature treatment. **(B)** Taxon evenness for communities at each temperature treatment. The black dots and the error bars represent the mean and the SEM. The gray dots are the values obtained in each of the 20 community replicates. The red line represents the fit of a unimodal model (see details of model selection in Supplementary Table S3).

**Figure 4:**
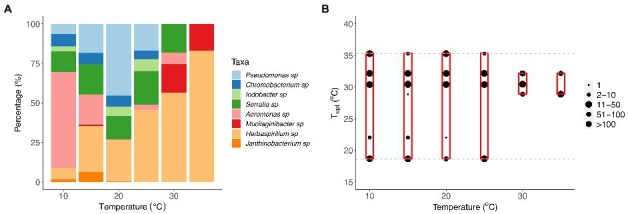
Changes in community composition with temperature. **(A)** Composition of bacterial taxa within communities incubated at 10, 15, 20, 25, 30, and 35°C for 7 days. Bar plot representing the percentage (%) of each taxon recovered in the samples at each temperature after the incubation period. **(B)** The composition of optimal growth temperatures for bacterial taxa within communities incubated at 10, 15, 20, 25, 30, and 35°C for 7 days. The gray dashed lines represent the optimal temperatures of bacterial taxa that were present in the original eight-taxa communities, and the red boxes represent the range of optimal temperatures of the taxa recovered within each temperature treatment at the end of the experiment. The size of the point represents the number of colonies of each taxon within the communities present on agar plates after incubation.

To investigate the effect of temperature on community structure and composition and the role of taxon-specific thermal traits on community composition, we assembled artificial communities with the eight taxa previously characterized along a thermal gradient (10–35°C). After 1 week, we quantified the composition and relative abundance of each taxon within each community. Indices of taxon richness and evenness were significantly affected by temperature (richness: *F*_2,111_ = 9.75, *r*^2^ = 0.15, *p* < 0.001; evenness: *F*_2,87_ = 4.57, *r*^2^ = 0.1, *p* = 0.01, Figure 3; Supplementary Table S3). The effects of temperature on taxon richness were best characterized by a unimodal model in which richness remained relatively unchanged between 10 and 30°C, but then declined dramatically at 35°C, where only two species made up the community (*Herbaspirillum* sp. and *Mucilaginibacter sp.*), in which *Herbaspirillum sp*. dominated (>80%; Figure 4A; Supplementary Figure S1). Evenness also exhibited a unimodal relationship with temperature, being best characterized by a quadratic model, where equitability in taxon abundance peaks at intermediate temperatures 15–25°C and declined significantly at the extremes (10 and 35°C). These patterns were reflected in the rank abundance curves, which were steepest at the coldest (10°C) and hottest (35°C) temperatures, while shallower slopes were observed at intermediate temperatures (15–30°C; Supplementary Figure S1), highlighting that abundance was concentrated in a small number of highly abundant species at extreme temperatures.

To quantify whether species composition also changed with temperature, we performed a principal component analysis. We found significant differences in the composition of taxa present at the end of the experiment among the six temperature treatments (Supplementary Figure S2, PERMANOVA: *F*_1,112_ = 39.61, *p* = 0.001, Supplementary Table S8). Pairwise comparisons showed that there was a significant difference in community composition between the communities incubated at 10 and 35°C and all other communities (see Supplementary Table S9). *Janthinobacterium*, *Chromobacterium*, *Iodobacter*, and *Serratia sp.* were present within communities exposed at intermediate temperatures (Figure 4A; Supplementary Figure S2). *Pseudomonas sp.* was more persistent within communities at 20°C and *Aeromonas sp*. dominated at 10°C, whereas *Mucilaginibacter* and *Herbaspirillum sp.* were associated with the higher temperature treatments (30 and 35°C; Supplementary Figure S2).

**Figure 5:**
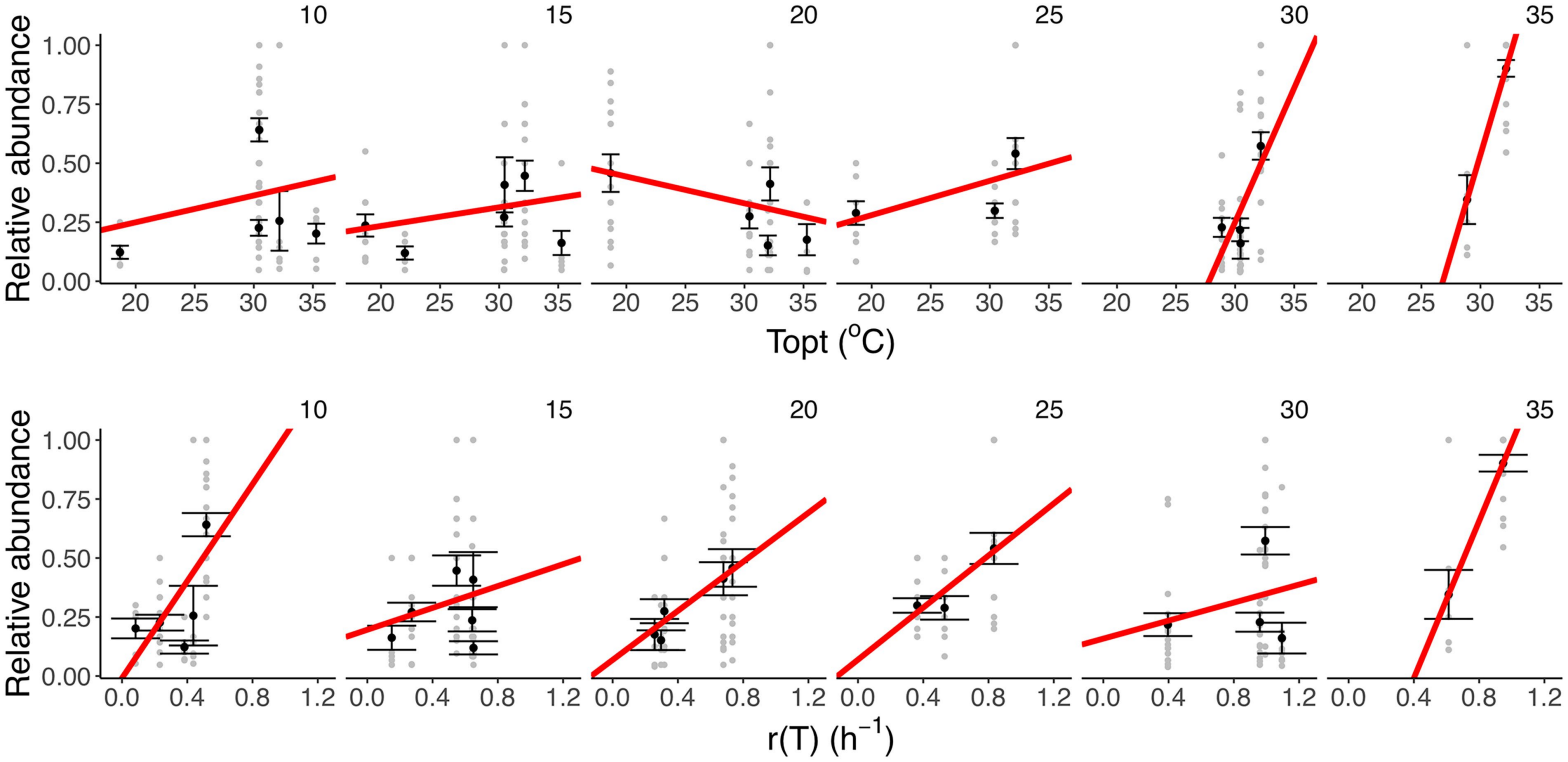
Linking taxon-specific thermal traits with relative abundance. Upper row: thermal optima (*T*_opt,_°C). Lower row: growth rate at the assay temperature [*r*(T), h^−1^]. Each panel represents a temperature treatment. Gray dots represent the relative abundance and taxon-specific thermal trait in each replicate community. The black dots and the error bars represent the averaged relative abundance per taxon and the SEM. The red lines represent the fit of a linear model (see details of model selection in Supplementary Table S4). We found a marked increase in the strength of the correlation between *T*_opt_ and relative abundance in the high-temperature treatments (30 and 35°C), while for *r* (T) we observe a positive relationship in the slope at all temperature treatments (35°C).

To assess whether taxon-specific thermal traits were predictive of community composition, we isolated the different taxa at each temperature and quantified their relative abundance at the end of the experiment. We found that the optimum temperature was significantly correlated with the relative abundance of the taxa within communities at different temperatures (*F*_11,283_ = 11.55, *r*^2^ = 0.31, *p* < 0.001), particularly the high-temperature treatments (30 and 35°C; Figures 4B, 5; Supplementary Table S4). We also found that growth rate at the test temperature was significantly correlated to the relative abundance of the taxa within communities across the thermal gradient (*F*_11,283_ = 12.5, *r*^2^ = 0.33, *p* < 0.001; Figure 5; Supplementary Table S4).

**Figure 6:**
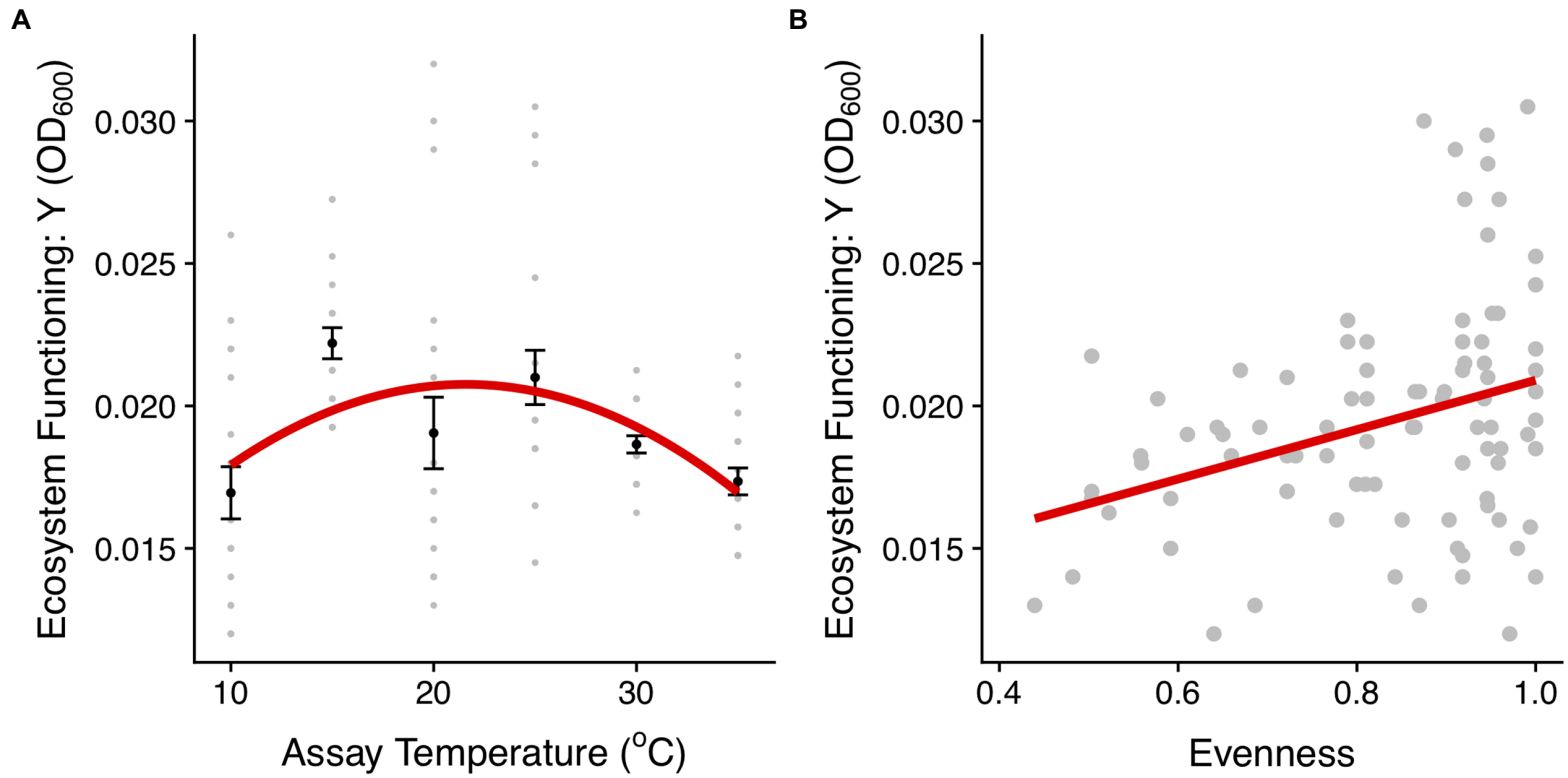
Linking community structure and ecosystem functioning. **(A)** Biomass production (OD_600_) for communities in each temperature treatment. The red line represents the fit of a quadratic model (see details of model selection in Supplementary Table S7). The black dots and the error bars represent the mean across replicates for each temperature treatment and the SEM. The gray points are the different community replicates for each temperature treatment. **(B)** Relationship between the evenness in the abundance distribution and ecosystem functioning estimated using the asymptotic biomass (OD_600_). The red lines represent the fit of a linear model (*p* < 0.01, Supplementary Table S7).

To understand how changes in community structure affect the productivity of the communities along the thermal gradient, we compared community biomass (OD_600_), as an estimate of ecosystem functioning, with the evenness of taxon abundance. Both evenness and ecosystem functioning (biomass accumulated) exhibited quadratic relationships with temperature (evenness: *F*_2,87_ = 4.57, *r*^2^ = 0.1, *p* = 0.01; ecosystem function: *F*_2,117_ = 7.62, *r*^2^ = 0.12, *p* < 0.001, Figures 3B, 6; Supplementary Tables S3, S7). Communities with higher evenness were more productive which tended to correspond with intermediate temperatures (mainly 25°C). Indeed, we found a significant correlation between evenness and biomass across the experiment (*F*_1,88_ = 10.46, *r*^2^ = 0.11, *p* = 0.002, Figure 6B).

## Discussion

We assembled communities of eight bacterial isolates that express wide variation in thermal performance (Figure 2; Supplementary Table S2) and exposed them to a broad gradient in temperature to investigate how variance in thermal tolerance traits between taxa shape structural and functional changes in microbial communities under different thermal regimes. We hypothesized that temperature-driven changes in taxonomic composition, community structure, and ecosystem function would be linked to taxon-specific thermal traits (Figure 1). Our experimental approach aimed to understand the processes that determine how environmental change drives community reorganization and ecosystem functioning.

At the community level, we observed marked changes in the number of species present at the end of the experiment but also in the abundance distribution with temperature. As we initially hypothesized, at intermediate temperatures (Figure 1, Case 2, 15–25°C), we found greater evenness in the distribution of abundance (Figure 3; Supplementary Figure S1). However, as temperature departed from ambient conditions either *via* cooling (Figure 1, Case 1, 10°C) or warming (Figure 1, Case 3, 30 and especially 35°C), evenness decreased (Figure 3; Supplementary Figure S1) and communities became highly skewed, being dominated by a small number of taxa (Figure 4A; Supplementary Figure S1). In addition to the changes in the distribution of biomass among taxa caused by temperature change, we also observed a decline in species richness with increasing temperature, especially beyond 25°C (Figure 3; Supplementary Figure S1).

Our results also revealed significant taxonomic differences in community composition along the thermal gradient (Figure 4; Supplementary Figure S2). These compositional differences were particularly pronounced between the extreme temperature treatments (10, 30, and 35°C) and the rest of the assay temperatures (Supplementary Table S9). We also found that some taxa were associated with these extreme temperatures; for example, *Aeromonas* sp. was associated with 10°C, *Mucilaginibacter sp.* with 30°C, and *Herbaspirillum sp.* with 35°C (Figure 4; Supplementary Figure S2). We also found that *Pseudomonas sp.* was the dominant species at ambient temperature (see Figure 4; Supplementary Figure S2, 20°C). To explore any links between changes in community composition and the taxon-specific thermal traits, we compared the relative abundance of taxa in each community with the *T*_opt_ of the taxa measured when grown in monoculture. In line with our expectations, we found that the compositional and structural changes were linked to variation in *T*_opt_, as evidenced by the significant correlation between *T*_opt_ and the relative abundance of each taxon (Figure 5). The correlation between *T*_opt_ and relative abundance was particularly pronounced at the high-temperature treatments (30 and 35°C; Figure 5; Supplementary Table S5). We observed that the taxa present in the low diversity, 30 and 35°C treatments, were within the group of taxa exhibiting high optimum temperatures for growth (28.9–32.2°C) but also higher growth rates at the test temperatures in monoculture (Figures 2, 5; Supplementary Figure S2). Notably, no taxa with optimal growth temperatures below 28.9°C were present at the end of the experiment at these temperatures (Figure 4B).

While taxon-specific thermal optimum was a good predictor of community structure and function, particularly at high temperatures, there were anomalies that could not be explained by this thermal trait alone. For example, *Chromobacterium* sp., which had the highest thermal optimum of 35.3°C, was not present in communities incubated at 30 and 35°C. However, despite *Chromobacterium sp.* being the taxon with the highest *T*_opt_, it did not have the highest performance (growth rate) at those temperatures. The taxa (e.g., *Herbaspirillum* and *Mucilaginibacter sp*) with higher growth rates were therefore able to outcompete *Chromobacterium sp.* and dominate community biomass at 30 and 35°C (Figures 2, 4). This result emphasizes that differences in growth rate as well as thermal optima are important traits for understanding how microbial community dynamics respond to temperature change.

Having established the impacts of temperature on microbial community structure and composition, we then investigated the links between community structure and ecosystem functioning along the thermal gradient. We found that like evenness in the abundance distribution, community biomass production (functioning) followed a unimodal relationship with temperature peaking at intermediate temperatures and declining at the extremes (Figure 6A). Asymptotic biomass was therefore positively correlated with evenness (Figure 6B), suggesting a link between the effect of warming on community structure and the emergent outcome for ecosystem functioning. The taxon-specific thermal tolerance curves imply that a large proportion of taxa are free from thermal stress at intermediate temperatures and thus temperature imposed very little environmental selection. By contrast, at high and low temperatures only a small number of taxa were able to tolerate these more extreme conditions (as evidenced by the taxon-specific thermal tolerance curves) and were able to dominate community abundance in the absence of competitors. Consequently, the more equitable abundance distribution and higher diversity at intermediate temperatures meant that a greater proportion of the total niche space available could be occupied by the species and the community was able to more fully exploit available resources and yield higher total biomass (Tilman, 2004).

While taxon-specific thermal traits were a good predictor of community composition and function at temperature extremes, they provided far less explanatory power at intermediate temperatures. When temperatures are benign for most taxa, i.e., at temperatures lower than thermal optima but higher than temperatures that cause low-temperature stress, temperature-driven selection is likely to be weak relative to other factors that limit growth, such as resource availability. Under these benign thermal conditions, thermal traits, which characterize fitness at the bounds of environmental tolerance, are expected to be of little utility to understanding the dynamics of microbial community turnover and instead other traits that capture how fitness is impacted by the limiting environmental factor, i.e., resource acquisition traits, will play a predominant role. It is therefore important to stress that while a useful part of the ecologist’s arsenal, thermal tolerance traits have significant limitations to understanding microbial community dynamics outside of the specific cases where temperatures exceed thermal tolerance limits for taxa within regional species pool.

Understanding how rising temperatures will affect community structure and composition is crucial in predicting the impact of warming on ecosystem services. Our multi-level experiment demonstrated the links between taxon-specific thermal traits and the effects of temperature on community structure and ecosystem functioning. Our results highlight that a simple trait from thermal tolerance curves, such as the optimum temperature for growth and the rate of growth at the test temperature, offers significant explanatory power in understanding how aspects of community structure and function respond to warming. These results add to a large body of recent work highlighting how phenotypic traits offer a valuable conceptual and theoretical bridge that can link population, community, and ecosystem approaches to develop a mechanistic understanding of ecosystem-level responses to environmental change (Norberg et al., 2001; Lavorel and Garnier, 2002; Hooper et al., 2006; McGill et al., 2006; Litchman and Klausmeier, 2008; Enquist et al., 2015).

## Data availability statement

The original contributions presented in the study are included in the article/Supplementary material, further inquiries can be directed to the corresponding authors.

## Author contributions

FCG and GY-D designed research, analyzed the data, and wrote the paper. FCG and RW performed research. All authors contributed to the article and approved the submitted version.

## Funding

This work was supported by a European Research Council (ERC) grant awarded to GY-D (ERC StG 677278 TEMPDEP).

## Conflict of interest

The authors declare that the research was conducted in the absence of any commercial or financial relationships that could be construed as a potential conflict of interest.

The reviewer DK declared a past collaboration with one of the authors GY-D to the handling editor.

## Publisher’s note

All claims expressed in this article are solely those of the authors and do not necessarily represent those of their affiliated organizations, or those of the publisher, the editors and the reviewers. Any product that may be evaluated in this article, or claim that may be made by its manufacturer, is not guaranteed or endorsed by the publisher.
