# Supplementary material for: Thermal traits govern the response of microbial community dynamics and ecosystem functioning to warming
Source: Front Microbiol. 2022 Aug 17;13:906252. doi: 10.3389/fmicb.2022.906252 (PMC9428465; doi:10.3389/fmicb.2022.906252)
Supplement: Supplementary file 1 [file Data_Sheet_1.pdf]

## Supplementary Material

**Title:** Thermal traits govern the response of microbial community dynamics and ecosystem functioning to warming

**Running title:** Thermal traits and community dynamics

**Authors:** *Francisca C. García[1*], Ruth Warfield[1], Gabriel Yvon-Durocher[1]*

**Affiliation:**

[1]Environment and Sustainability Institute, University of Exeter, Penryn, Cornwall TR10 9EZ, UK

*** Correspondence to:** paquigrcgrc@gmail.com, G.Yvon-Durocher@exeter.ac.uk

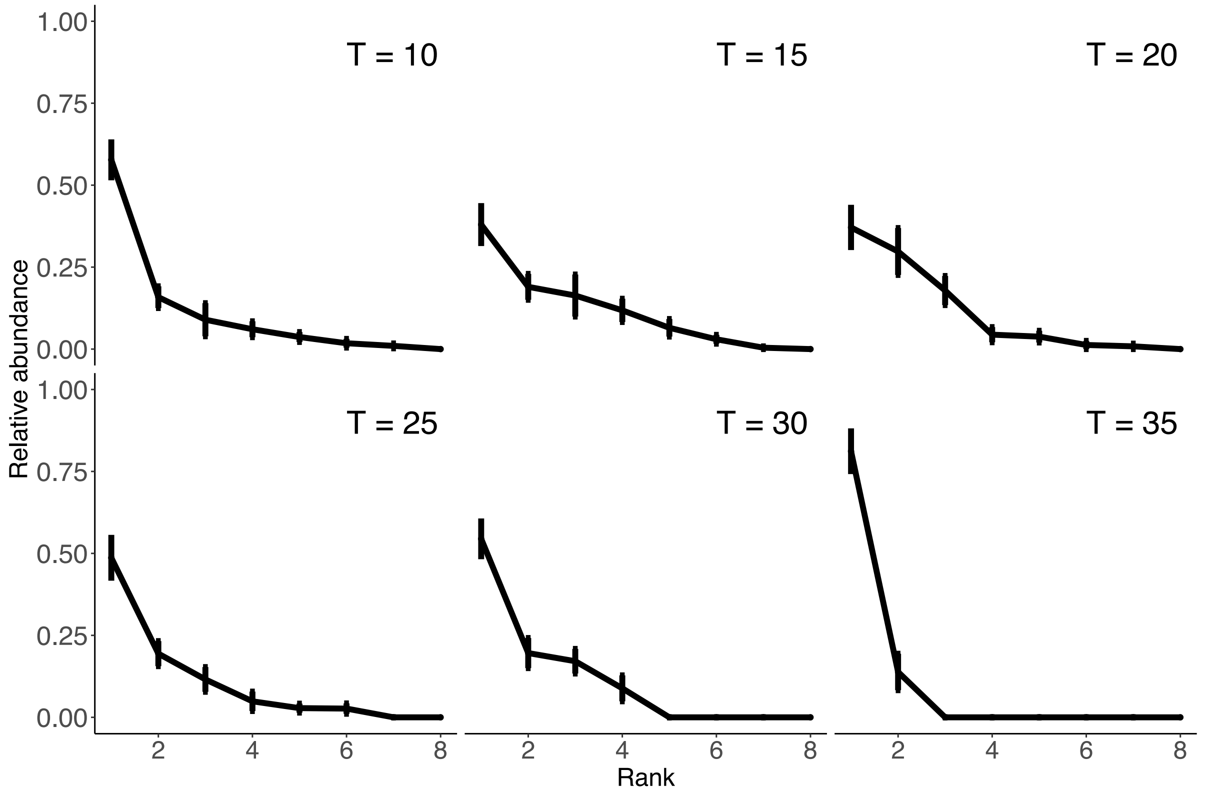


**Fig S1. Changes in community structure with temperature**. Rank abundance curves for the communities incubated at 10 °C, 15 °C, 20 °C, 25 °C, 30 °C and 35 °C. The dots represent the mean abundance of each taxon and the error bars illustrate the standard error of the mean (S.E.M.) across 20 replicates. We found that communities incubated at extreme temperatures had steepest rank abundance curves implying lower species richness and evenness.

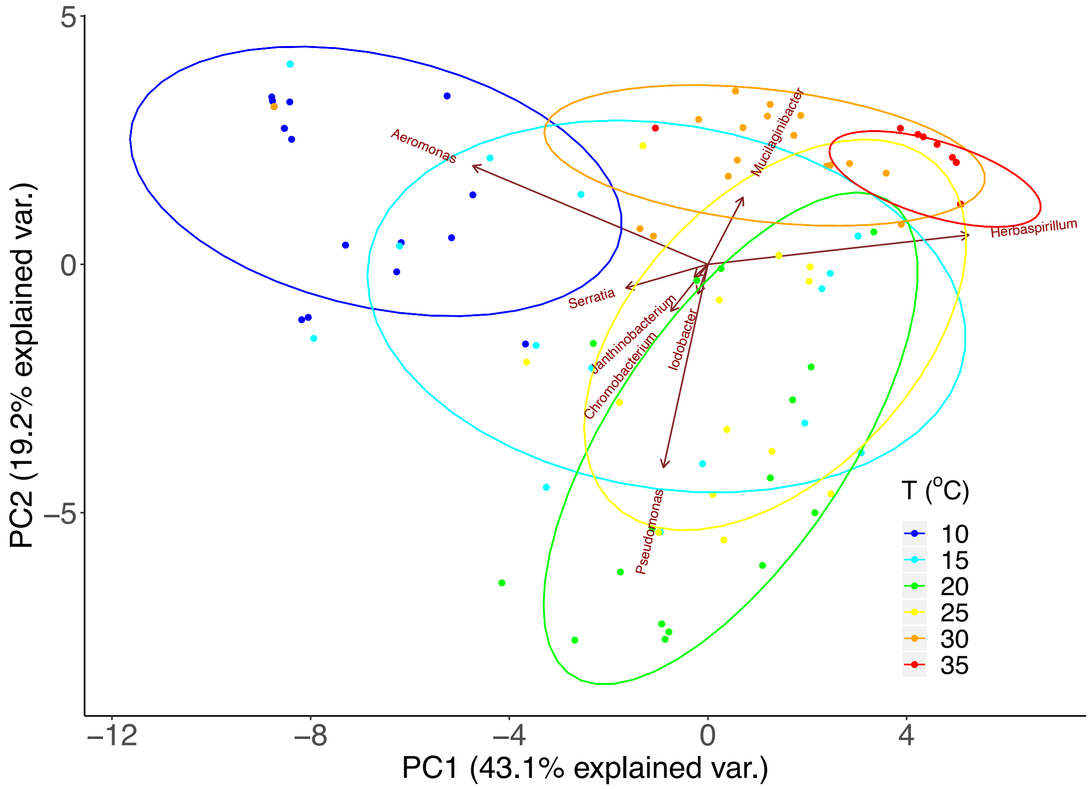


**Fig S2.** Principal components analysis of taxonomic composition within microbial communities grown along the thermal gradient. The brown arrows signify the correspondence of each species with the primary axes of variation (species scores). The coloured points represent each replicate community in the various temperature treatments and its correspondence with the axes of variation (site scores). Coloured ellipses show the 95% confidence interval around the centroid of each temperature treatment. Non-overlapping ellipses suggest significant divergence in community composition between the temperature treatments.

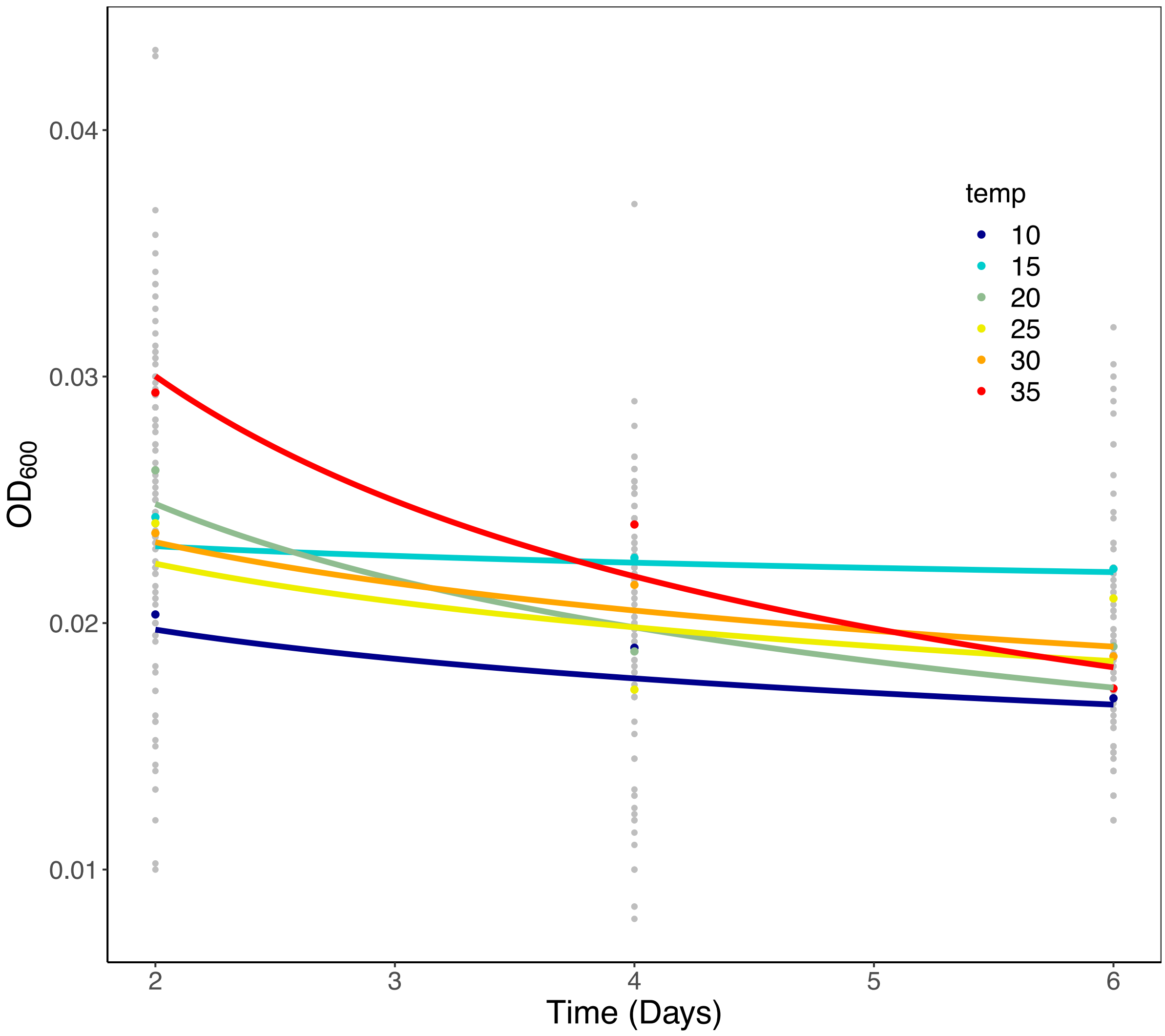


**Fig S3.** Community biomass dynamics. Changes in community biomass ($OD_{600}$) during the incubation time of the across the thermal gradient. The grey dots are the 20 replicates. The solid colour dots represent the average biomass at each temperature treatment and sampling day and the solid lines represents the fit of a logarithmic function fitted to the data via a mixed effect model (see Table S10 for details)

**Table S1. List of bacterial isolates used in the experiment.** Isolate identification codes. Taxonomic information, stream ID, average daily water temperature at the time of sampling (°C) and GenBank accession numbers are given.

| Isolate ID | Genus | Family | Phylum | Stream | Temp stream | Accession number |
|---|---|---|---|---|---|---|
| w_Ic161A | *Pseudomonas sp* | Pseudomonaceae | Proteobacteria | 1A | 8 | MZ506751 |
| n_Ic175L | *Chromobacterium sp* | Neisseriaceae | Proteobacteria | 5L | 19 | MZ506749 |
| p_Ic175L | *Iodobacter sp* | Neisseriaceae | Proteobacteria | 5L | 19 | MZ506750 |
| h_Ic174 | *Serratia sp* | Enterobacteriaceae | Proteobacteria | 4 | 10 | MZ506746 |
| n_Ic167 | *Aeromonas sp* | Aeromonadaceae | Proteobacteria | 7 | 11.4 | MZ506748 |
| d_Ic171 | *Mucilaginibacter sp* | Sphingobacteriaceae | Bacteroidetes | 1 | 17 | MZ506744 |
| j_Ic165 | *Herbaspirillum sp* | Oxalobacteraceae | Proteobacteria | 5 | 26.9 | MZ506747 |
| h_Ic161A | *Janthinobacterium sp* | Oxalobacteraceae | Proteobacteria | 1A | 8 | MZ506745 |

**Table S2: Parameters from the Sharpe-Schoolfield equation fitted to growth rate data to derive th thermal tolerance curve for each taxon**. $E_a$ represents the activation energy (eV), $E_h$ the deactivation energy (eV), the temperature where half the enzymes are rendered non-functional ($T_h$), $T_{\text{opt}}$ the optimum temperature (°C), $R(T_c)$ is the rate of growth normalised to an arbitrary reference temperature, $T_c$ = 18 ºC. The quasi $r^2$ represents the quality of the model fitting. The Akaike Information Criterion (AIC) of the model fitting is provided. The 95% confident intervals (CI) for each parameter estimated using Sharpe- Schoofield equation are given.

| Isolate ID | ln $(r(Tc))$ | | $E_a$ | | $E_h$ | | $T_h$ | | $T_{opt}$ | AIC | quasi $r^2$ |
|---|---|---|---|---|---|---|---|---|---|---|---|
| w_Ic161A | 0.26 | (-4.63,5.16) | 1.01 | (-3.97,6.00) | 2.11 | (-2.22,6.44) | 18.96 | (263.39,320.82) | 18.67 | 48.20 | 0.52 |
| n_Ic175L | -1.55 | (-1.84,-1.26) | 0.82 | (-0.46,1.17) | 1.98 | (0.52,3.45) | 36.77 | (299.98,319.87) | 35.28 | 22.5 | 0.92 |
| p_Ic175L | -1.32 | (-1.85,-0.80) | 0.45 | (-0.08,0.97) | 2.89 | (1.37,4.41) | 36.78 | (301.72,318.13) | 31.98 | 33.62 | 0.80 |
| h_Ic174 | -1.20 | (-1.73,-0.68) | 0.23 | (-0.30,0.76) | 2.57 | (0.85,4.29) | 37.82 | (300.75,321.20) | 30.42 | 33.45 | 0.78 |
| n_Ic167 | -0.29 | (-1.24,0.66) | 0.32 | (-0.70,1.35) | 2.37 | (-0.41,5.14) | 36.79 | (289.31,330.57) | 30.46 | 45.08 | 0.39 |
| d_Ic171 | -0.53 | (-1.25,0.19) | 0.48 | (-0.33,1.28) | 2.86 | (1.40,4.31) | 33.37 | (-296.25,316.79) | 28.87 | 39.25 | 0.81 |
| j_Ic165 | -0.46 | (-1.21,0.28) | 0.32 | (-0.38,1.01) | 3.43 | (1.07,5.79) | 37.63 | (301.50,320.05) | 32.17 | 40.89 | 0.76 |
| h_Ic161A | 0.09 | (-1.40,1.57) | 1.06 | (-0.75,2.88) | 2.63 | (1.11,4.16) | 23.13 | (284.95,307.61) | 22.02 | 38.43 | 0.85 |

**Table S3: Details of the model analysing the effects of temperature on species richness and evenness.** The results of the Analysis of variance (ANOVA) for the model selection procedure on the fixed effect terms are given and the most parsimonious models are highlighted in bold. The effects of temperature on species richness and evenness were best characterised by the quadratic model provided the best fit (model 2) . Analyses reveal that both richness (R) and evenness (J) changed significantly through the different temperature treatments. The residual degrees of freedom (d.f.), residual sum of squares (RSS), Sum of squares (SumSqs), F value and p-value are given.

| **Model** | **Res.d.f** | **RSS** | **SumSqs** | **F** | **p** |
|---|---|---|---|---|---|
| Richness (R) | | | | | |
| model structure | | | | | |
| 1. R~ 1 + T | 112 | 199.57 | | | |
| **2. R ~ T + I($T^2$)** | **111** | **185.51** | **14.062** | **8.41** | **0.004 **** |
| Evenness (J) | | | | | |
| model structure | | | | | |
| 1. J ~ 1 + T | 88 | 2.02 | | | |
| **2. J ~ T + I($T^2$)** | **87** | **1.88** | **0.16** | **7.72** | **0.006 **** |

**Table S4. Analysis of covariance (ANCOVA) comparing the relationship between community composition and taxon-specific traits at different assay temperatures.** Analyses reveal that relative abundance was significantly linked to the traits of the species (taxon-specific thermal optima ($T_{opt}$, °C) and taxon-specific growth rate at each assay temperature ($r$(T), $h^{-1}$)) and that the slope and intercept were significantly different between assay temperatures. The degrees of freedom (d.f.), sum of squares (Sum Sqs), mean of squares (Mean Sqs), F- value and p-value are given. The estimated parameters, the 95% confident intervals, p-value, number of observations and squared r are also provided for both ANCOVA models.

| **Model** | **d.f** | **SumSqs** | **MeanSqs** | **F** | **p** |
|---|---|---|---|---|---|
| Response: Relative abundance (RA)<br>RA~ $T_{opt}$ * T | | | | | |
| $T_{opt}$ | 1 | 0.49 | 0.49 | 8.32 | <0.01** |
| *T* | 5 | 3.70 | 0.74 | 12.52 | <0.001*** |
| $T_{opt}$:T | 5 | 3.32 | 0.66 | 11.24 | <0.001*** |
| Residuals | 283 | 16.73 | 0.06 | - | - |
| Response: Relative abundance (RA)<br>RA~ $r$ (T) * T | | | | | |
| $r$(T) | 1 | 3.04 | 3.04 | 52.65 | <0.001*** |
| *T* | 5 | 3.38 | 0.68 | 11.72 | <0.001*** |
| $r$(T):T | 5 | 1.52 | 0.30 | 5.26 | <0.001*** |
| Residuals | 283 | 16.32 | 0.06 | - | - |

| **Parameters** | | **$T_{opt}$** | |
|---|---|---|---|
| *Predictors* | *Estimates* | *CI* | *p* |
| (Intercept) | 0.02 | - 0.44 – 0.48 | 0.924 |
| Topt | 0.01 | - 0.00 – 0.03 | 0.145 |
| T [15] | 0.05 | - 0.51 – 0.62 | 0.853 |
| T [20] | 0.65 | 0.08 – 1.22 | **0.025** |
| T [25] | -0.03 | - 0.66 – 0.60 | 0.918 |
| T [30] | -3.18 | -4.77 – - 1.59 | **<0.001** |
| T [35] | -4.54 | -6.52 – - 2.56 | **<0.001** |
| Topt * T [15] | -0.00 | - 0.02 – 0.02 | 0.720 |
| Topt * T [20] | -0.02 | -0.04 – - 0.00 | **0.019** |
| Topt * T [25] | 0.00 | - 0.02 – 0.02 | 0.769 |
| Topt * T [30] | 0.10 | 0.05 – 0.15 | **<0.001** |
| Topt * T [35] | 0.16 | 0.09 – 0.22 | **<0.001** |
| Observations | 295 | | |

| **Parameters** | | **$r$(T)** | |
|---|---|---|---|
| *Predictors* | *Estimates* | *CI* | *p* |
| (Intercept) | 0.62 | 0.47 – 0.76 | **<0.001** |
| ln rate | 0.22 | 0.11 – 0.33 | **<0.001** |
| T [15] | -0.22 | -0.41 – -0.04 | **0.019** |
| T [20] | -0.10 | -0.29 – 0.10 | 0.328 |
| T [25] | -0.04 | -0.24 – 0.16 | 0.698 |
| T [30] | -0.26 | -0.43 – -0.10 | **0.002** |
| T [35] | 0.35 | 0.16 – 0.55 | **<0.001** |
| ln rate * T [15] | -0.12 | -0.28 – 0.04 | 0.146 |
| ln rate * T [20] | 0.02 | -0.16 – 0.21 | 0.800 |
| ln rate * T [25] | 0.09 | -0.15 – 0.32 | 0.477 |
| ln rate * T [30] | -0.09 | -0.27 – 0.09 | 0.351 |
| ln rate * T [35] | 1.06 | 0.58 – 1.53 | **<0.001** |
| Observations | 295 | | |

| | | | |
|---|---|---|---|
| $R^2$ / $R^2$ adjusted | 0.310/ 0.283 | $R^2$ / $R^2$ adjusted | 0.327 / 0.301 |

**Table S5. Multiple comparison contrast analysis for the relationship between relative abundance and taxon-specific thermal optimum at different assay temperatures.** Data shown are the contrast analysis for the relative abundance – thermal optimum ($T_{opt}$, °C) relationship at different temperatures. This analysis tests whether the slope and the intercept are significantly different between all pairwise combinations of temperature treatments. The standard error (SE), t value and p-value were estimated for all possible pairwise combinations.

| Linear Hypotheses | Estimate | SE | t value | p-value |
|---|---|---|---|---|
| Intercept.T10 vs. Intercept.T15 == 0 | 0.08 | 0.17 | 0.45 | 0.998 |
| Intercept.T10 vs. Intercept.T20 == 0 | 0.67 | 0.17 | 4.03 | <0.001 *** |
| Intercept.T10 vs. Intercept.T25 == 0 | -0.01 | 0.22 | -0.05 | 1.00 |
| Intercept.T10 vs. Intercept.T30 == 0 | -3.15 | 0.77 | -4.08 | <0.001 *** |
| Intercept.T10 vs. Intercept.T35 == 0 | -4.52 | 0.98 | -4.63 | <0.001 *** |
| Intercept.T15 vs. Intercept.T20 == 0 | 0.60 | 0.24 | 2.52 | 0.10 |
| Intercept.T15 vs. Intercept.T25 == 0 | -0.09 | 0.28 | -0.31 | 1 |
| Intercept.T15 vs. Intercept.T30 == 0 | -3.23 | 0.79 | -4.08 | <0.001 *** |
| Intercept.T15 vs. Intercept.T35 == 0 | -4.59 | 0.99 | -4.64 | <0.001 *** |
| Intercept.T20 vs. Intercept.T25 == 0 | -0.68 | 0.28 | -2.48 | 0.12 |
| Intercept.T20 vs. Intercept.T30 == 0 | -3.83 | 0.79 | -4.84 | <0.001 *** |
| Intercept.T20 vs. Intercept.T35 == 0 | -5.19 | 0.99 | -5.24 | <0.001 *** |
| Intercept.T25 vs. Intercept.T30 == 0 | -3.15 | 0.80 | -3.91 | <0.01 ** |
| Intercept.T25 vs. Intercept.T35 == 0 | -4.51 | 1.00 | -4.51 | <0.001 *** |
| Intercept.T30 vs. Intercept.T35 == 0 | -7.72 | 1.33 | -5.80 | <0.001 *** |
| Sc.T10 vs. Sc.T15 == 0 | 0.008 | 0.006 | 1.40 | 0.70 |
| Sc.T10 vs. Sc.T20 == 0 | -0.01 | 0.006 | -2.00 | 0.310 |
| Sc.T10 vs. Sc.T25 == 0 | 0.01 | 0.007 | 0.95 | 0.34 |
| Sc.T10 vs. Sc.T30 == 0 | 0.11 | 0.03 | 4.49 | <0.001 *** |
| Sc.T10 vs. Sc.T35 == 0 | 0.17 | 0.03 | 5.38 | <0.001 *** |
| Sc.T15 vs. Sc.T20 == 0 | -0.02 | 0.008 | -2.40 | 0.14 |
| Sc.T15 vs. Sc.T25 == 0 | -0.007 | 0.009 | -0.71 | 0.98 |
| Sc.T15 vs. Sc.T30 == 0 | 0.11 | 0.03 | 4.08 | <0.001 *** |
| Sc.T15 vs. Sc.T35 == 0 | 0.16 | 0.03 | 5.05 | <0.001 *** |
| Sc.T20 vs. Sc.T25 == 0 | 0.03 | 0.009 | 2.76 | 0.06 |
| Sc.T20 vs. Sc.T30 == 0 | 0.12 | 0.03 | 4.82 | <0.001 *** |
| Sc.T20 vs. Sc.T35 == 0 | 0.18 | 0.03 | 5.65 | <0.001 *** |
| Sc.T25 vs. Sc.T30 == 0 | 0.10 | 0.03 | 3.76 | <0.01 ** |
| Sc.T25 vs. Sc.T35 == 0 | 0.15 | 0.03 | 4.78 | <0.001 *** |
| Sc.T30 vs. Sc.T35 == 0 | 0.05 | 0.04 | 1.40 | 0.73 |

**Table S6. Multiple comparison contrast analysis for the relationship between relative abundance and taxon-specific growth rate at different assay temperatures.** Data shown are the contrast analysis for the relative abundance – growth rate ($r$ (T), $h^{-1}$) relationship at different temperatures. This analysis tests whether the slope and the intercept are significantly different between all pairwise combinations of temperature treatments. The standard error (SE), t value and p-value were estimated for all possible pairwise combinations.

| Linear Hypotheses | Estimate | SE | t value | p-value |
|---|---|---|---|---|
| Intercept.T10 vs. Intercept.T15 == 0 | 0.20 | 0.08 | 2.41 | 0.19 |
| Intercept.T10 vs. Intercept.T20 == 0 | 0.07 | 0.09 | 0.75 | 0.99 |
| Intercept.T10 vs. Intercept.T25 == 0 | 0.07 | 0.12 | 0.60 | 1.00 |
| Intercept.T10 vs. Intercept.T30 == 0 | 0.16 | 0.09 | 1.69 | 0.62 |
| Intercept.T10 vs. Intercept.T35 == 0 | -0.67 | 0.26 | -2.60 | 0.12 |
| Intercept.T15 vs. Intercept.T20 == 0 | -0.13 | 0.12 | -1.04 | 0.95 |
| Intercept.T15 vs. Intercept.T25 == 0 | -0.13 | 0.14 | -0.89 | 0.98 |
| Intercept.T15 vs. Intercept.T30 == 0 | -0.04 | 0.12 | -0.30 | 1.00 |
| Intercept.T15 vs. Intercept.T35 == 0 | -0.87 | 0.27 | -3.20 | 0.03 * |
| Intercept.T20 vs. Intercept.T25 == 0 | 0.001 | 0.15 | 0.007 | 1.00 |
| Intercept.T20 vs. Intercept.T30 == 0 | 0.09 | 0.13 | 0.69 | 1.00 |
| Intercept.T20 vs. Intercept.T35 == 0 | -0.74 | 0.28 | -2.70 | 0.1 |
| Intercept.T25 vs. Intercept.T30 == 0 | 0.09 | 0.15 | 0.59 | 1.00 |
| Intercept.T25 vs. Intercept.T35 == 0 | -0.74 | 0.28 | -2.61 | 0.12 |
| Intercept.T30 vs. Intercept.T35 == 0 | -0.83 | 0.28 | -3.02 | 0.04 * |
| Sc.T10 vs. Sc.T15 == 0 | -1.82 | 0.47 | -3.91 | <0.01 ** |
| Sc.T10 vs. Sc.T20 == 0 | -1.53 | 0.47 | -3.29 | 0.02 * |
| Sc.T10 vs. Sc.T25 == 0 | -1.50 | 0.47 | -3.17 | 0.03 * |
| Sc.T10 vs. Sc.T30 == 0 | -1.86 | 0.45 | -4.13 | <0.001 *** |
| Sc.T10 vs. Sc.T35 == 0 | -0.39 | 0.53 | -0.73 | 0.99 |
| Sc.T15 vs. Sc.T20 == 0 | 0.29 | 0.23 | 1.27 | 0.87 |
| Sc.T15 vs. Sc.T25 == 0 | 0.32 | 0.24 | 1.33 | 0.84 |
| Sc.T15 vs. Sc.T30 == 0 | -0.04 | 0.19 | -0.21 | 1.00 |
| Sc.T15 vs. Sc.T35 == 0 | 1.43 | 0.34 | 4.19 | <0.001 *** |
| Sc.T20 vs. Sc.T25 == 0 | 0.03 | 0.24 | 0.13 | 1.00 |
| Sc.T20 vs. Sc.T30 == 0 | -0.33 | 0.19 | -1.70 | 0.61 |
| Sc.T20 vs. Sc.T35 == 0 | 1.14 | 0.34 | 3.34 | 0.02 * |
| Sc.T25 vs. Sc.T30 == 0 | -0.36 | 0.21 | -1.72 | 0.59 |
| Sc.T25 vs. Sc.T35 == 0 | 1.11 | 0.35 | 3.16 | 0.03 * |
| Sc.T30 vs. Sc.T35 == 0 | 1.47 | 0.32 | 4.60 | <0.001 *** |

**Table S7. Details of the model selection analysis quantifying the effect of temperature (T) and evenness (J) on Ecosystem functioning (EF).** The results of the Analysis of variance (ANOVA) for the model selection procedure on the fixed effect terms are given and the most parsimonious models are highlighted in bold. For temperature, model selection revealed that a quadratic relationship between temperature on ecosystem function provided the best fit. Conversely, for eveness, the best fitting model implied a linear relationship between evenness and ecosystem functioning. Analyses reveal that both temperature and evenness have a significant effect on ecosystem functioning. The residual degrees of freedom (d.f.), residual sum of squares (RSS), Sum of squares (SumSqs), F value and p-value are given.

| **Model** | **Res.d.f** | **RSS** | **SumSqs** | **F** | **p** |
|---|---|---|---|---|---|
| Ecosystem functioning (EF) | | | | | |
| model structure | | | | | |
| 1. EF ~ 1 + T | 118 | 0.002 | | - | - |
| **2. EF ~ T + I($T^2$)** | **117** | **0.002** | **$0.2*10^{-3}$** | **14.35** | **<0.001 *****|
| Ecosystem functioning (EF) | | | | | |
| model structure | | | | | |
| **1. EF ~ 1 + J** | **88** | **0.001** | | - | - |
| 2. EF~ J + I($J^2$) | 87 | 0.001 | $3.82*10^{-7}$ | 0.03 | 0.087 |

**Table S8: Results from the Permutational Multivariate Analysis of Variance (PERMANOVA) to test the effect of temperature on community composition.** The table shows the parameters obtained from Permutational Multivariate Analysis of Variance (PERMANOVA) test to analyse whether there is significant difference between the species composition at each temperature treatment.

| Parameter | d.f | Sum of Sqs | Mean Sqs | F-value | $R^2$ | p-value |
|---|---|---|---|---|---|---|
| Temperature | 1 | 6.46 | 6.46 | 39.61 | 0.26 | <0.001 (***) |
| Residuals | 112 | 18.27 | 0.16 | | 0.74 | |
| Total | 113 | 24.73 | | | 1 | |

**Table S9: Results from the pairwise comparison of the Permutational Multivariate Analysis of Variance (PERMANOVA) to test the effect of temperature on community composition.** The table shows the parameter obtained from the pairwise Permutational Multivariate Analysis of Variance (PERMANOVA) comparison to test whether there are significant differences between all pairwise combinations of the temperature treatments. p is the p-value and p.adjusted is the adjusted p value using Bonferroni correction.

| Temperature | F | $R^2$ | p-value | p.adjusted |
|---|---|---|---|---|
| 10-15 | 13.47 | 0.27 | 0.001 | 0.02 * |
| 10-20 | 25.67 | 0.42 | 0.001 | 0.02 * |
| 10-25 | 32.7 | 0.48 | 0.001 | 0.02 * |
| 10-30 | 33.68 | 0.48 | 0.001 | 0.02 * |
| 10-35 | 74.13 | 0.67 | 0.001 | 0.02 * |
| 15-20 | 2.47 | 0.06 | 0.06 | 0.80 |
| 15-25 | 1.94 | 0.05 | 0.13 | 1 |
| 15-30 | 5.08 | 0.12 | 0.002 | 0.045 * |
| 15-35 | 16.28 | 0.31 | 0.001 | 0.02 * |
| 20-25 | 2.34 | 0.06 | 0.09 | 1 |
| 20-30 | 9.91 | 0.21 | 0.001 | 0.02 * |
| 20-35 | 19.8 | 0.35 | 0.001 | 0.02 * |
| 25-30 | 5.16 | 0.13 | 0.004 | 0.02* |
| 25-35 | 16.33 | 0.32 | 0.001 | 0.02 * |
| 30-35 | 10.68 | 0.22 | 0.001 | 0.02 * |

**Table S10. Details of the linear mixed effects model analysis for the change in community biomass ($OD_{600}$) with time (t) at different assay temperatures (T). We fit a linear model between natural log transformed biomass and time, refelecting the logarithmic decline in biomass through time in the experiment.** Random effects on intercept were determined at the level of replicates. The results of the model selection procedure on the fixed effect terms are given and the most parsimonious model is highlighted in bold. Analyses reveal that community biomass followed a decellerating logarithmic decline with time and that the exponent and intercept were significantly different between selection temperatures. The degrees of freedom (d.f.), AIC, logLik, Chi square (Chisq) and p-value are given.

| **Model** | **d.f** | **AIC** | **Log Lik** | **Chisq** | **p** |
|---|---|---|---|---|---|
| Biomass (OD) | | | | | |
| Random effects structure | | | | | |
| ~1/Rep:T + 0 + ln t \| Rep:T | | | | | |
| Fixed effects structure | | | | | |
| 1. ln OD ~ ln *t* + T | 10 | -14.54 | 17.27 | - | - |
| **2. ln OD ~ ln *t* * T** | **15** | **-33.99** | **31.99** | **29.45** | **< 0.0001 *****|
